# Neonatal Chylothoraces: A 10-Year Experience in a Tertiary Neonatal Referral Centre

**DOI:** 10.1155/2019/3903598

**Published:** 2019-03-13

**Authors:** Marie K. White, Ravindra Bhat, Anne Greenough

**Affiliations:** ^1^Neonatal Intensive Care Centre, Kings College Hospital NHS Foundation Trust, Denmark Hill, London SE5 9RS, UK; ^2^Department of Women and Children's Health, School of Life Course Sciences, Faculty of Life Sciences and Medicine, King's College London, London SE5 9RS, UK; ^3^Asthma UK Centre for Allergic Mechanisms in Asthma, King's College London, London SE5 9RS, UK; ^4^NIHR Biomedical Research Centre at Guy's and St. Thomas' NHS Foundation Trust and King's College London, Guy's Hospital, Great Maze Pond, London SE1 9RT, UK

## Abstract

**Background:**

Neonatal chylothorax is a rare condition, but has a high mortality.

**Study Objectives:**

To analyse the outcomes of a series of neonates with chylothorax and review the literature to determine best practice.

**Design:**

A case series review and a literature review using electronic databases including the key words neonates and chylothorax.

**Results:**

Six cases of neonatal chylothorax were identified during a ten-year period, two had congenital chylothoraces and four iatrogenic chylothoraces after thoracic surgery or chest instrumentation. The neonates were ventilated for a median of 30 (range 13–125) days with a median maximum daily pleural fluid output of 218 (range 86–310) ml/kg/day. All the neonates were given medium-chain triglyceride (MCT) feeds which stabilised pleural fluid output in four and reduced it in another. Octreotide was used in three neonates, but the dosage used had no significant effect on pleural output. Two neonates required surgical intervention. The literature review demonstrated MCT feeds can reduce or stabilise pleural fluid output, but highlighted variable use of octreotide and inconsistent dosing regimens and outcomes. No consensus regarding indications for surgical intervention was identified.

**Summary and Conclusion:**

Neonatal chylothorax is uncommon, but affected neonates require high healthcare utilisation.

## 1. Introduction

Neonatal chylothorax is a rare condition with an incidence of 1 in 5775 [1] to 24000 [2] and has a high mortality rate of up to 64% [2]. Neonatal chylothorax can be congenital or acquired, the latter most commonly occurs after damage to the thoracic duct during surgery [3]. We have reviewed our experience in a tertiary neonatal surgical unit and set this in the context of a literature review to establish if it was possible to determine best practice.

## 2. Case Series

### 2.1. Method

A retrospective study was undertaken to determine the outcomes of all neonates with neonatal chylothoraces admitted to a tertiary medical and surgical neonatal intensive care unit in London, UK, from July 2008 to July 2018. Neonates were identified through the hospital clinical coding and an electronic database (BadgerNet). The diagnosis of a neonatal chylothorax was made if the pleural fluid had a cell count of >1000/ml with a lymphocyte predominance and if the triglyceride level was greater than 1.1 mmol/L in a neonate who was being fed [4]. Initial treatment for our cohort included stabilisation, ventilatory support, and replacement of pleural fluid losses. Specific treatment included medium-chain triglyceride (MCT) feeds, and then octreotide if the pleural fluid output continued, followed by surgical intervention if those interventions failed.

A literature review was undertaken using electronic databases including the key words neonates and chylothorax.

### 2.2. Results

There were 54,488 births in the study period, and six neonates were diagnosed with chylothorax, giving a local overall incidence of 1 in 9081 births. The six neonates had a median gestation of 37 + 6 (range 33 + 5 to 39 + 6) weeks and a median birth weight of 2935 (range 2252–4078) grams (Table 1). All neonates had a “lymphocytic” effusion, and three had a raised pleural fluid triglyceride of 2.2 (range 1.7–3.2) mmol/L. Two neonates had genetic variants of unknown significance and one, with subtle dysmorphic features, had a benign copy variant of paternal inheritance.

Four neonates required intubation in the delivery suite, and all required an extended period of mechanical ventilation with a median duration of 30 (range 13–125) days (Table 1). Two neonates required high-frequency oscillation ventilation and inhaled nitric oxide. Five neonates were successfully extubated and self-ventilating in air by a median of 37 (range 30–78) days. The neonates required a median of five (range 2–6) chest drains for a median duration of 36 (range 28–99) days. The median maximum total daily pleural fluid output was 218 (range 86–310) ml/kg/day. The majority of pleural drain losses were replaced by human albumin solution (HAS) or occasionally by 0.9% saline or fresh frozen plasma (FFP).

All the neonates required total parental nutrition and were commenced on MCT feeds after diagnosis. Five achieved full MCT feeds at a median of 37 (range 11–44) days of age. One neonate had resolution of the chylothorax following introduction of MCT feeds. In four neonates, MCT feeds were associated with stabilisation of the pleural fluid output. Two neonates were graded back to either a standard neonatal formula or maternal expressed breast milk, and three were discharged on MCT feeds. One neonate had increased pleural fluid output after the introduction of MCT feeds which were subsequently stopped and the neonate remained on total parental nutrition until reorientation of care.

Three neonates were treated with a continuous octreotide intravenous infusion for a median of seven (range 6–16) days with a starting dose of 1-2 micrograms/kg/hr to a maximum of 2–8 micrograms/kg/hr. One neonate developed transient hypothyroidism and, therefore, remained on low-dose octreotide at 2 micrograms/kg/hr, which was stopped after thoracic duct ligation. Octreotide was stopped in the other two neonates as there was no reduction in pleural fluid output. Two neonates with acquired chylothorax required operative intervention. One had a successful thoracic duct ligation and one had bilateral pleurodesis, but this failed to improve the neonate's outcome. Five neonates survived, and the total length of stay until discharge or death was a median of 68 (range 44–127) days.

## 3. Discussion

Neonatal congenital chylothorax is a rare condition as evidenced by an incidence of 1 in 27,244 in our cohort, which is in keeping with another study (1 in 24,000 [2]). Our mortality (17%) is in accordance with other studies [1, 2, 12]. Up to 50% of neonates with neonatal chylothorax can be associated with genetic syndromes [1, 14]. In our cohort, although two neonates had genetic variants, none were found to have a defined syndrome.

In our cohort, only one neonate had a reduction in pleural fluid output following the introduction of MCT feeds, but in others the output was stabilised. MCT feeds have been shown to be effective in reducing lymphatic flow in 25% of neonates with surgically related neonatal chylothorax [15]. Indeed, our literature review (Table 2) demonstrated that the majority of neonates who have a neonatal chylothorax receive MCT feeds.

A systematic review in which the efficacy of octreotide, a somatostatin analogue, was assessed did not recommend its use [4]. Our literature review demonstrated since then multiple case series in which octreotide was used have been published with variable dosing regimens, length of treatment, and outcomes and that octreotide was not used in all neonates (Table 2) [1, 2, 7–14]. A recent systematic review [3] found octreotide to be effective in 47% of neonates with neonatal chylothorax. Many of the papers included, however, did not describe what constituted a clinical improvement or the timescale over which any changes occurred. In our cohort, there was no significant improvement in the pleural fluid output in the three neonates treated with octreotide. None of these neonates reached the dose of 10 micrograms/kg/hr which some studies have used. Multiple side effects have been reported in up to 14% neonates treated with octreotide including hyperglycaemia, necrotising enterocolitis, transient mild cholestasis, transient hypothyroidism, pulmonary hypertension, and severe hypotension [3]. In our cohort, one neonate developed transient hypothyroidism.

The literature review (Table 2) demonstrated only a minority of neonates required either pleurodesis or thoracic duct ligation. This is consistent with the findings of our cohort. A review of a case series of neonatal “surgical” chylothoraces mainly following congenital diaphragmatic hernia repair also demonstrated conservative management was successful in the majority [15]. It would be important to develop evidence-based guidelines as to when such interventions should be used.

## 4. Conclusions

Our results highlight neonatal chylothorax is an uncommon condition, but is associated with high healthcare utilisation. Neonates require stabilisation with ventilation, pleural drainage, and fluid replacement. MCT feeds can reduce and stabilise pleural fluid output. It is important to optimise the dose of octreotide and monitor for side effects. Multicentre trials are required to identify the optimum evidenced-based management for neonatal chylothorax. Although neonatal chylothorax is an uncommon condition, we believe that multicentre trials would be feasible as shown by the randomised trial of respiratory support in neonates with congenital diaphragmatic hernia [16].

## Figures and Tables

**Table 1:** Perinatal and neonatal characteristics, management, and outcomes.

Neonate	Birth weight (g)	Gestation (weeks)	Antenatal	Mode of delivery	Resuscitation	Diagnoses	MCT feeds	Octreotide and dose	Ventilation (days)	Outcome
1	3080	39	Normal	VD	Thick meconium intubated at 10 minutes	Acquired NC, MAS, multiple pneumothoraces	Yes	No	38	Alive
2	2252	33	Hydrops, bilateral effusions, antenatal shunts	VD	Pleural shunts clamped & intubated at birth	Congenital NC	Yes	Yes (2–8 mcg/kg/hr)	31	Alive
3	4078	38	Left pleural effusion & mediastinal shift	CS	Intubated at birth	Congenital NC	Yes	No	28	Alive
4	2790	37	Left CDH	VD	Intubated at birth	Acquired NC, CDH	Yes	Yes^*∗∗*^ (1–4 mcg/kg/hr)	125	Died
5	2690	38	Polyhydramnios	VD	No resuscitation	Acquired NC, OA, TOF	Yes	Yes^*∗∗*^ (1–2 mcg/kg/hr)	13	Alive
6	3350	37	Polyhydramnios	VD	No resuscitation	Acquired NC, OA, TOF	Yes^*∗*^	No	19	Alive

CDH: congenital diaphragmatic hernia; CS: caesarian section; MAS: meconium aspiration syndrome; MCT: medium-chain triglycerides; NC: neonatal chylothorax; OA: oesophageal atresia; TOF tracheoesophageal fistula; VD: vaginal delivery; ^*∗*^neonate had a reduction in pleural output following introduction of MCT feeds; ^*∗∗*^octreotide was given prior to surgical intervention.

**Table 2:** Case series of neonatal chylothorax.

Author and year	Number of neonates	Ventilation (days)	Number given MCT feeds	Number treated with octreotide and dose	Octreotide duration (days)	Octreotide efficacy	Pleurodesis or thoracic duct ligation
Altuncu et al. (2007) [5]	3	N/R	*n* = 3	*n* = 1 (1–10 mcg/kg/hr)	28	No response	*n* = 1
Matsukuma et al. (2009) [6]	2	N/R	*n* = 2	*n* = 2 (0.5–10 mcg/kg/hr)	16–20	No response	*n* = 2
Bellini et al. (2012) [7]	30	30	*n* = 28	*n* = 6 (1–10 mcg/kg/hr)	8–38	Decreased chylous production, but not quantified	*n* = 1
Horvers et al. (2012) [8]	7	16	*n* = 7	*n* = 7 (2–12 mcg/kg/hr)	Median 22 (11–46)	Possible response in two neonates	*n* = 0
Shah and Sinn (2012) [9]	6	30	*n* = 6	*n* = 6 (0.5–10 mcg/kg/hr)	Median 20 (4–41)	Resolution in five neonates	*n* = 0
Downie et al. (2013) [1]	10	8	*n* = 7	*n* = 3 (3.5–10 mcg/kg/hr)	N/R	Response in two neonates: not quantified	*n* = 0
Landis et al. (2013) [10]	11	11	N/R	*n* = 6 (1–13 mcg/kg/hr)	N/R	No response	*n* = 0
Bialkowski et al. (2015) [2]	28	23	*n* = 25	*n* = 7 (3–10 mcg/kg/hr)	Median 21 (10–45)	No response	*n* = 0
Hua et al. (2016) [11]	4	1	N/R	*n* = 3 (N/A)	14	No response	*n* = 4
Yin et al. (2017) [12]	14	13	*n* = 12	*n* = 3 (1–6 mcg/kg/hr)	Median 6	Average drain output significantly lower: three days after treatment. Median 62 ml versus 133 ml (*p*=0.002)	*n* = 0
Shillitoe et al. (2018) [13]	21	N/R	*n* = 2	*n* = 1 (maximum dose 10 mcg/kg/hr)	N/R	On the maximum dose there was a 50% reduction in five days	*n* = 0
Zaki et al. (2018) [14]	9	N/R	*n* = 9	*n* = 9 (1–10 mcg/kg/hr)	5–66	Resolution in three neonates: not quantified	*n* = 0

N/R: not reported.

## References

[1] Downie L., Sasi A., Malhotra A. (2014). Congenital chylothorax: associations and neonatal outcomes. *Journal of Paediatrics and Child Health*.

[2] Bialkowski A., Poets C. F., Franz A. R. (2015). Congenital chylothorax: a prospective nationwide epidemiological study in Germany. *Archives of Disease in Childhood Fetal Neonatal Edition*.

[3] Bellini C., Cabano R., De Angelis L. C. (2018). Octreotide for congenital and acquired chylothorax in newborns: a systematic review. *Journal of Paediatrics and Child Health*.

[4] Das A., Shah P. S. (2010). Octreotide of the treatment of chylothorax in neonates. *Cochrane Database of Systematic Reviews*.

[5] Altuncu E., Akman İ., Kıyan G. (2007). Report of three cases: congenital chylothorax and treatment modalities. *Turkish Journal of Pediatrics*.

[6] Matsukuma E., Aoki Y., Sakai M. (2009). Treatment with OK-432 for persistent congenital chylothorax in newborn infants resistant to octreotide. *Journal of Pediatric Surgery*.

[7] Bellini C., Ergaz Z., Radicioni M. (2012). Congenital fetal and neonatal visceral chylous effusions: neonatal chylothorax and chylous ascites revisited: a multicenter retrospective study. *Lymphology*.

[8] Horvers M., Mooij C. F., Antonius T. A. J. (2012). Is octreotide treatment useful in patients with congenital chylothorax. *Neonatology*.

[9] Shah D., Sinn J. K. (2012). Octreotide as therapeutic option for congenital idiopathic chylothorax: a case series. *Acta Paediatrica*.

[10] Landis M. W., Butler D., Lim F. Y. (2013). Octreotide for chylous effusions in congenital diaphragmatic hernia. *Journal of Pediatric Surgery*.

[11] Hua Q.-W., Lin Z.-Y., Hu X.-T., Zhao Q.-F. (2016). Treatment of persistent congenital chylothorax with intrapleural injection of sapylin in infants. *Pakistan Journal of Medical Science*.

[12] Yin R., Zhang R., Wang J. (2017). Effects of somatostatin/octreotide treatment in neonates with congenital chylothorax. *Medicine*.

[13] Shillitoe B. M. J., Berrington J., Athiraman N. (2018). Congenital pleural effusions: 15 years single-centre experience from North-East England. *Journal of Maternal-Fetal & Neonatal Medicine*.

[14] Zaki S. A., Krishnamurthy M. H., Malhotra A. (2018). Octreotide use in neonates: a case series. *Drugs in R&D*.

[15] Costa K. M., Saxena A. K. (2018). Surgical chylothorax in neonates: management and outcomes. *World Journal of Pediatrics*.

[16] Snoek K. G., Capolupo I., van Rosmalen J. (2016). Conventional mechanical ventilation versus high-frequency oscillatory ventilation for congenital diaphragmatic hernia. *Annals of Surgery*.

